# Integration of ATAC-Seq and RNA-Seq Reveals VDR–SELENBP1 Axis Promotes Adipogenesis of Porcine Intramuscular Preadipocytes

**DOI:** 10.3390/ijms252312528

**Published:** 2024-11-22

**Authors:** Jiawei Zhou, Junjing Wu, Tao Yang, Xinyu Zhang, Mu Qiao, Zhong Xu, Yu Zhang, Yue Feng, Tong Chen, Zipeng Li, Xianwen Peng, Shuqi Mei

**Affiliations:** 1 Institute of Animal Science and Veterinary Medicine, Hubei Academy of Agricultural Sciences, Wuhan 430064, China; zhoujiawei@hbaas.com (J.Z.); wujunjing@hbaas.com (J.W.); 15565054903@163.com (T.Y.); z1289348438@163.com (X.Z.); qiaomu@hbaas.com (M.Q.); xuzhong@hbaas.com (Z.X.); zhangyu@hbaas.com (Y.Z.); fengyue@hbaas.com (Y.F.); ctokay@163.com (T.C.); lizipeng@hbaas.com (Z.L.); 2 College of Animal Science and Technology, Huazhong Agricultural University, Wuhan 430070, China

**Keywords:** pig, intramuscular fat, accessible chromatin, *SELENBP1*, adipogenic differentiation

## Abstract

Intramuscular fat (IMF) content plays a crucial role in determining pork quality. Recent studies have highlighted transcriptional mechanisms controlling adipogenesis in porcine IMF. However, the changes in chromatin accessibility during adipogenic differentiation are still not well understood. In this study, we performed the assay for transposase-accessible chromatin with high-throughput sequencing (ATAC-seq) and transcriptome sequencing (RNA-Seq) analyses on porcine intramuscular preadipocytes to explore their adipogenic differentiation into mature adipocytes. We identified a total of 56,374 differentially accessible chromatin peaks and 4226 differentially expressed genes at day 0 and day 4 during adipogenic differentiation. A combined analysis of the ATAC-seq and RNA-seq data revealed that 1750 genes exhibited both differential chromatin accessibility and differential RNA expression during this process, including selenium-binding protein 1 (*SELENBP1*), *PLIN1*, *ADIPOQ*, and *FASN*. Furthermore, we found that vitamin D receptor (VDR) could bind to the promoter region of the *SELENBP1* gene, activate *SELENBP1* transcription, and ultimately promote lipid accumulation during adipogenic differentiation. This study provides a detailed overview of chromatin accessibility and gene expression changes during the adipogenic differentiation of porcine intramuscular preadipocytes. Moreover, we propose a novel regulatory mechanism involving the VDR–SELENBP1 signaling axis in adipogenic differentiation.

## 1. Introduction

The intramuscular fat (IMF) content is a critical factor in determining pork quality, as it plays a significant role in enhancing meat taste and flavor [1]. Due to the anatomical and physiological similarities between humans and pigs, pigs are increasingly recognized as valuable biomedical models for human health studies [2]. IMF accumulation is not only relevant for pork quality but is also associated with various health conditions, such as diabetes, insulin resistance, and cardiovascular disease [3,4,5]. Therefore, elucidating the molecular mechanisms that regulate IMF development in pigs is vital for both improving meat quality and advancing our understanding of human health-related adipogenesis.

Adipose tissue consists of various cell types, including adipocytes, preadipocytes, mesenchymal stem cells, fibroblasts, and immune cells [6]. Adipogenesis, the process of fat cell formation, occurs in two phases: the commitment phase, where adipose precursor cells are generated, and the differentiation phase, which leads to adipocyte maturation [7]. A key signaling pathway involved in adipogenic differentiation includes the sequential activation of transcription factors such as cAMP-response element-binding protein (CREB), CCAAT/enhancer-binding protein (C/EBP)-β, and C/EBPδ. C/EBPβ activation promotes the expression of peroxisome proliferator activated receptor γ (*PPARγ*) and *C/EBPα*, both of which are crucial for initiating adipogenesis [8]. *PPARγ* and *C/EBPα* further regulate the expression of genes essential for adipocyte development [9]. Transcription factor PATZ1 promotes adipogenesis through its interaction with the transcriptional machinery at the promoter regions of essential early adipogenic factors [10]. Thus, adipogenesis in vivo requires specific spatiotemporal transcriptional activities to occur effectively.

Chromatin accessibility pertains to the degree to which chromatin DNA interacts with regulatory elements [11]. Numerous adipogenic transcription factors co-occupy hotspots characterized by an open chromatin structure and specific epigenetic modifications; these transcription factor hotspots serve as essential signaling centers that integrate diverse adipogenic signals at specific chromatin sites, thereby facilitating a coordinated regulation of gene expression [12,13]. The assay for transposase-accessible chromatin with high-throughput sequencing (ATAC-seq) is a technique widely used to identify open chromatin regions and predict transcription factors involved in gene regulation [14,15]. Recently, combining ATAC-seq with transcriptome sequencing (RNA-seq) has been instrumental in uncovering the regulatory mechanisms governing tissue development in pigs [16,17]. For example, these combined methodologies have been applied to study the longissimus dorsi muscle (LDM) tissue in different pig breeds, leading to the identification of key cis-regulatory elements that influence fat production, such as Mef2c, C/EBP, TFAP4, MAX, and NHLH1 [18,19]. Studies have highlighted variations in chromatin accessibility and gene transcription regulation among pig species with varying IMF content. However, despite these advances, the LDM tissue, which is predominantly composed of muscle cells, may not adequately capture the unique aspects of adipogenesis in pigs with varying IMF content.

In the present study, we cultured intramuscular preadipocytes isolated from the LDM of 3-day-old piglets. By performing both ATAC-seq and RNA-seq analyses, we aimed to elucidate changes in chromatin accessibility and gene expression patterns occurring during adipogenic differentiation. This study seeks to enhance our understanding of the molecular mechanisms underlying IMF development, contributing to strategies for enhancing pork quality and identifying potential therapeutic targets for obesity.

## 2. Results

### 2.1. Identification of Porcine Intramuscular Preadipocytes During Adipogenic Differentiation

**Figure 1:**
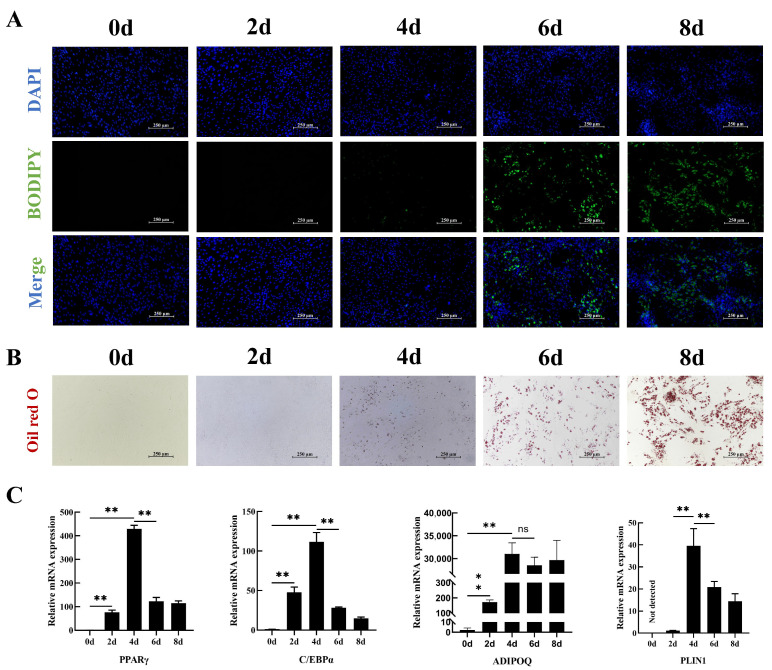
Induction of adipogenic differentiation of porcine intramuscular preadipocytes. (**A**) BODIPY and (**B**) Oil Red O staining of the preadipocytes at 0, 2, 4, 6, and 8 days of differentiation. (**C**) *PPARγ*, *C*/*EBPα*, *ADIPOQ*, and *PLIN1* mRNA levels in the preadipocytes during differentiation. ** *p* < 0.01, ns = non-significant.

To evaluate the in vitro differentiation potential of porcine intramuscular preadipocytes into adipocytes, we investigated lipid droplet formation during adipogenic differentiation. The findings from BODIPY and Oil Red O staining demonstrated that lipid droplets began to appear on day 4 of differentiation (Figure 1A,B and Figure S1). Concurrently, the expression levels of key adipogenic markers, including *PPARγ*, *C/EBPα*, and adiponectin (*ADIPOQ*), increased markedly, reaching their highest levels on day 4 of induction (*p* < 0.01; Figure 1C). These observations suggest that adipogenic differentiation is most transcriptionally active on the fourth day of differentiation.

### 2.2. Dynamics of Chromatin Accessibility During Adipogenic Differentiation

**Figure 2:**
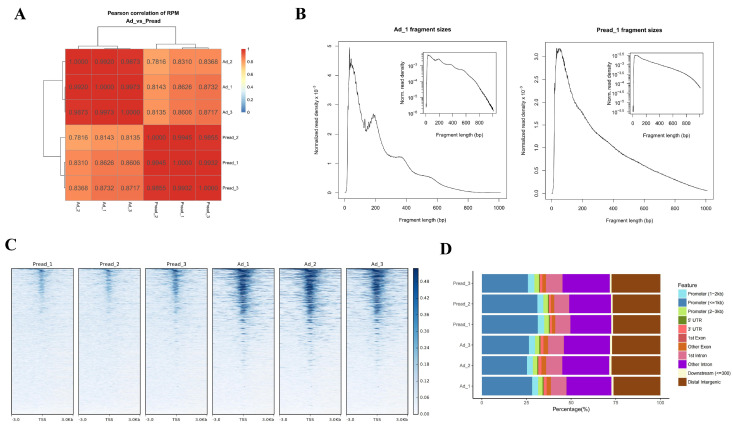
Overview of the ATAC-seq results. (**A**) The results of Pearson correlation analysis. (**B**) Fragment length distribution map. (**C**) A heatmap of the peak signals across the gene body of the library; ±3.0 represents upstream and downstream of the TSS. (**D**) Genomic distribution of the peaks in each sample.

To explore the mechanisms of transcriptional regulation during adipogenic differentiation in porcine intramuscular preadipocytes, chromatin accessibility was assessed at day 0 (Preadipocyte, Pread) and day 4 (Adipocyte, Ad) by utilizing ATAC-seq technology. This analysis generated a total of 41,588,977–49,397,933 raw reads per sample, of which 40,947,178–48,753,889 clean reads were uniquely aligned to the Sus scrofa 11.1 reference genome after data filtration (Table 1). The Pearson correlation coefficient analysis indicated a high level of similarity among replicates, whereas significant differences were observed between cells at different stages of differentiation (Figure 2A). The quality of the libraries was assessed through an analysis of the lengths of the inserted fragments and the distribution of peak signals. The examination of fragment sizes revealed that the predominant majority of fragments were less than 200 base pairs in length, which included one mononucleosome fragment and one nucleosome-free fragment. This finding suggests that all libraries are suitable for subsequent experimental procedures (Figure 2B). Most peaks were mapped to gene promoter regions within ±3 kb of transcription start sites (TSSs) (Figure 2C). Annotation of the average chromatin peaks using the reference genome revealed that a large proportion of these peaks were located within promoter regions, introns, and distal intergenic areas (Figure 2D).

**Figure 3:**
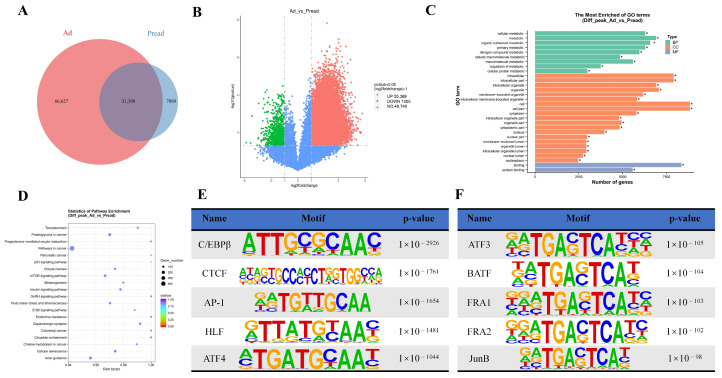
Identification and analysis of differentially accessible chromatin regions (DARs). (**A**) Diagram illustrates the overlap of peaks between the preadipocyte (Pread) and adipocyte (Ad) groups. (**B**) A volcano plot of differential peaks. (**C**) GO terms and (**D**) KEGG pathway enrichment analysis of DAR-associated genes. (**E**) Enriched transcription-factor-binding motifs identified through ATAC-seq for increased peaks between the Pread and Ad groups, and (**F**) motifs associated with the decreased peaks. * *p* < 0.05.

A total of 66,627 peaks specific to the Ad group, 7089 peaks specific to the Pread group, and 31,398 peaks common to both groups were identified (Figure 3A). Differential chromatin accessibility analysis using DiffBind revealed that adipocytes compared with preadipocytes exhibited 55,369 upregulated (more accessible) peaks and 1005 downregulated (less accessible) peaks (Figure 3B). Annotation of these differential peaks indicated that the upregulated peaks corresponded to 20,198 genes, whereas the downregulated peaks were associated with 822 genes (Table S1). The Gene Ontology (GO) term analysis unveiled that these genes were significantly enriched in pathways related to cellular metabolism, lipid metabolism, and fat cell differentiation (Figure 3C, Table S2). The Kyoto Encyclopedia of Genes and Genomes (KEGG) pathway analysis revealed the involvement of these genes in key pathways, including endocrine resistance, insulin signaling, and AMP-activated protein kinase (AMPK) signaling (Figure 3D, Table S3). We utilized the HOMER package to analyze motifs on different peaks of the Ad group and Pread group. Motif enrichment analysis of the peak regions highlighted significant enrichment of C/EBPβ, CTCF, AP-1, HLF, and ATF4 in the upregulated peaks (Figure 3E), while ATF3, BATF, FRA1, FRA2, and JunB were enriched in the downregulated peaks (Figure 3F).

### 2.3. Transcriptional Profiling of Porcine Intramuscular Preadipocytes During Adipogenic Differentiation

**Figure 4:**
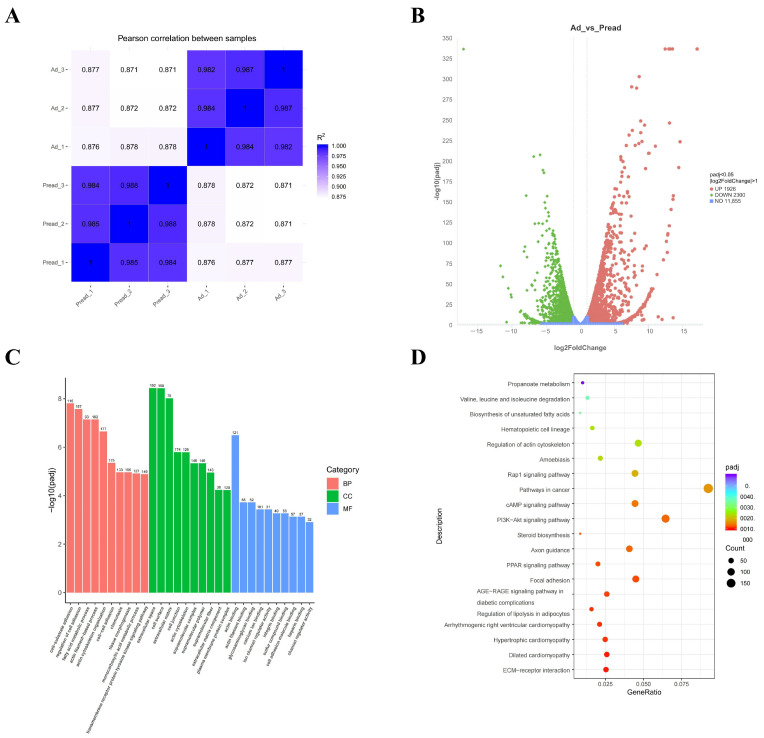
Analyses of RNA-seq. (**A**) The results of Pearson correlation analysis. (**B**) A volcano plot of DEGs. (**C**) GO terms and (**D**) KEGG enrichment analyses of DEGs.

To investigate the gene expression profiles associated with adipogenic differentiation, we performed RNA-seq on selected cells at day 0 and day 4 of adipogenic induction. In total, 72,053,888–85,485,102 high-quality reads were generated from the per sample libraries (Table 2). The Pearson correlation analysis unveiled strong correlations among replicates within each group, demonstrating the reliability of the data (Figure 4A). The differential expression analysis identified 4226 differentially expressed genes (DEGs), comprising 1926 upregulated and 2300 downregulated genes (Figure 4B, Table S4). Subsequently, GO and KEGG enrichment analyses of these DEGs revealed significant involvement in biological processes, such as cell-substrate adhesion, fatty acid metabolism, and actin filament-based (Figure 4C, Table S5). Additionally, the KEGG pathway analysis indicated that these DEGs were implicated in key pathways, including ECM-receptor interaction, the PPAR signaling pathway, and the regulation of lipolysis in adipocytes (Figure 4D, Table S6). Collectively, these data suggest that the identified DEGs play critical roles in regulating adipogenic differentiation. To assess the reliability of the RNA-seq data, eight genes associated with adipogenesis were chosen from the DEGs (*FABP4*, *CIDEC*, *PPARα*, *MGLL*, *LIPE*, *PNPLA2*, *DGAT2*, and *FASN*) for qRT-PCR analysis. The findings indicated that the expression patterns of these genes were congruent between the RNA-seq and qRT-PCR results (Figure S2).

### 2.4. Integration Analysis of ATAC-Seq and RNA-Seq

**Figure 5:**
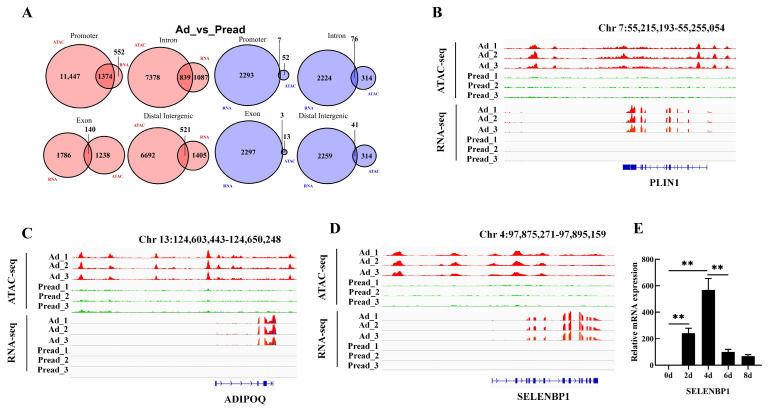
Integrative analyses of ATAC-seq and RNA-seq data. (**A**) Overlap between DARs and DEGs. (**B**) ATAC-seq and RNA-seq signals for *PLIN1*, (**C**) *ADIPOQ*, and (**D**) *SELENBP1* genes were determined through IGV. (**E**) The *SELENBP1* mRNA level in porcine intramuscular preadipocytes at 0, 2, 4, 6, and 8 days of adipogenic differentiation. ** *p* < 0.01.

To further understand the relationship between chromatin accessibility and gene expression during adipogenic differentiation, we performed an integrative analysis of the RNA-seq and ATAC-seq datasets. Our comparative analysis identified a total of 1750 genes showing overlap between the two datasets, which included 1628 concurrently upregulated genes and 122 concurrently downregulated genes (Table S7). Venn analysis of differential chromatin accessibility at promoter regions revealed that 1374 upregulated genes exhibited increased chromatin accessibility in these regions, whereas only seven downregulated genes showed decreased peak activity in these promoter regions (Figure 5A). In a previous study, we identified 56 proteins with increased expression in the high-IMF group compared to the low-IMF group in Xidu black pigs through proteomic analysis [20]. Of them, 12 proteins corresponded with the 1374 genes in our current analysis, including perilipin 1 (*PLIN1*), *ADIPOQ*, fatty acid synthase (*FASN*), and selenium-binding protein 1 (*SELENBP1*). Visual analysis using IGV software confirmed that both chromatin accessibility and transcription levels of *PLIN1*, *ADIPOQ*, and *SELENBP1* were significantly higher in the Ad group than in the Pread group (Figure 5B–D). Quantitative PCR validated that *SELENBP1* expression was markedly upregulated by day 4 of adipogenic differentiation, following an expression pattern similar to that of *PPARγ* and *C/EBPα* (*p* < 0.01; Figure 5E). Furthermore, GO term and KEGG pathway enrichment analyses of the genes co-upregulated in both ATAC-seq and RNA-seq datasets revealed significant enrichment in pathways related to lipid metabolism, fatty acid metabolism, and PPAR signaling pathway (Figure S3, Tables S8 and S9). These findings underscore the critical role of transcriptional regulation in adipogenic differentiation.

### 2.5. SELENBP1 Regulates Adipogenesis of Porcine Intramuscular Preadipocytes

**Figure 6:**
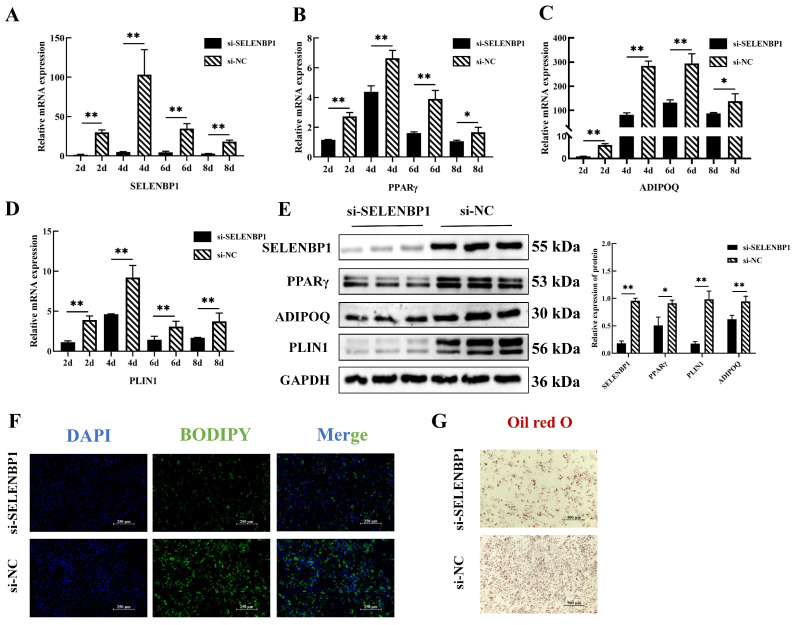
*SELENBP1* promotes adipogenesis of porcine intramuscular preadipocytes. (**A**) The mRNA expression levels of *SELENBP1*, (**B**) *PPARγ*, (**C**) *ADIPOQ*, and (**D**) *PLIN1* at 2, 4, 6, and 8 days of adipogenic differentiation following siRNA-SELENBP1 transfection into porcine intramuscular preadipocytes. (**E**) *SELENBP1*, *PPARγ*, *ADIPOQ*, and *PLIN1* protein levels following siRNA-SELENBP1 transfection into preadipocytes at 4 days. (**F**) BODIPY and (**G**) Oil Red O staining after siRNA-SELENBP1 transfection into preadipocytes at 4 days. * *p* < 0.05, ** *p* < 0.01.

To further investigate the regulatory role of *SELENBP1* in adipogenesis, we transfected intramuscular preadipocytes, isolated from 3-day-old piglets, with siRNA targeting *SELENBP1* (si-SELENBP1). This knockdown notably decreased *SELENBP1* expression during adipocyte differentiation at various time points (2, 4, 6, and 8 days). Along with the reduction in *SELENBP1*, we observed a marked downregulation of key adipogenic markers, including *PPARγ*, *ADIPOQ*, and *PLIN1* (*p* < 0.01; Figure 6A–E). Furthermore, BODIPY and Oil Red O staining demonstrated that *SELENBP1* knockdown significantly reduced lipid droplet formation, indicating impaired lipid accumulation in these cells (Figure 6F,G). These findings suggest that *SELENBP1* is crucial for the differentiation of porcine intramuscular preadipocytes and lipid accumulation during adipogenesis.

### 2.6. Transcription Factor Vitamin D Receptor Can Promote SELENBP1 Expression

**Figure 7:**
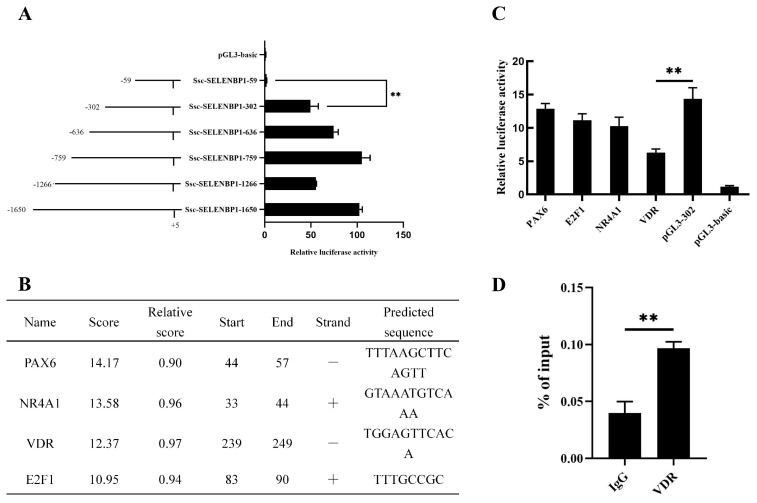
Identification of binding of VDR to *SELENBP1* promoter regions. (**A**) Luciferase assays were performed to detect the activities of a series of deletion constructs in porcine intramuscular preadipocytes. Luciferase activity was analyzed at 4 days after adipogenic differentiation. (**B**) JASPAR software (v2024) predicted the transcription-factor-binding sites located −302 bp to −59 bp upstream of the *SELENBP1* transcription start site. (**C**) Point mutations in the PAX6, VDR, NR4A1, and E2F1 binding sites of the *SELENBP1* promoter were analyzed through luciferase assays. (**D**) ChIP-qPCR results demonstrated that VDR could bind to the *SELENBP1* promoter region in porcine intramuscular preadipocytes at 4 days of differentiation. IgG was used as negative controls. ** *p* < 0.01.

Although our ATAC-seq analyses identified three significantly upregulated chromatin accessibility peaks in the promoter region of *SELENBP1* following adipogenic induction, the specific transcriptional mechanisms that regulate *SELENBP1* remain unclear. To elucidate these mechanisms, we performed luciferase reporter assays using constructs containing potential promoter regions of *SELENBP1*. Subsequent luciferase activity assays conducted in intramuscular preadipocytes indicated that the *pGL3-SELENBP1-302* construct (spanning −302 bp to −59 bp) was critical for *SELENBP1* transcriptional activity (*p* < 0.01; Figure 7A). To identify transcription factors that may bind to this core promoter region, we used the JASPAR (v2024) transcription factor prediction software. This analysis revealed potential binding sites for PAX6, NR4A1, vitamin D receptor (VDR), and E2F1 within the −302 bp to −59 bp region (Figure 7B). We performed luciferase activity assays with mutated binding sites for each transcription factor. Notably, mutations in the VDR binding site resulted in a significant reduction in promoter activity (*p* < 0.01; Figure 7C). By contrast, mutations in the other binding sites did not significantly affect activity. Chromatin immunoprecipitation (ChIP) analysis further confirmed that VDR directly binds to the promoter region of SELENBP1 (*p* < 0.01; Figure 7D). These findings indicate that VDR directly interacts with the SELENBP1 promoter, promoting its transcriptional activity.

### 2.7. VDR Regulates Adipogenesis of Porcine Intramuscular Preadipocytes via SELENBP1

**Figure 8:**
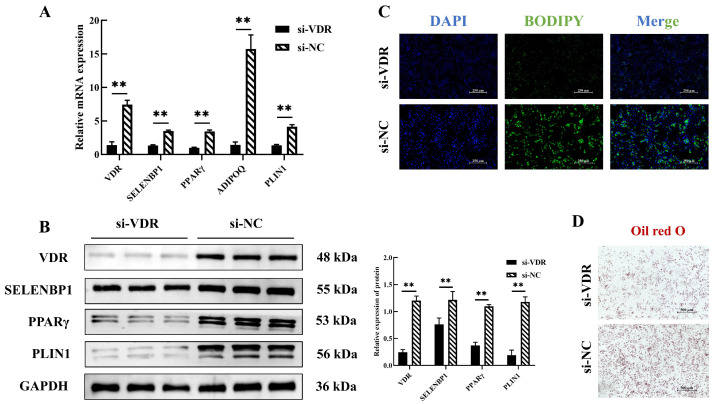
VDR promotes adipogenesis of porcine intramuscular preadipocytes by regulating *SELENBP1*. (**A**) *VDR*, *SELENBP1*, *PPARγ*, *ADIPOQ*, and *PLIN1* mRNA levels following siRNA-VDR transfection into porcine intramuscular preadipocytes at 4 days. (**B**) VDR, SELENBP1, PPARγ, and PLIN1 protein levels following siRNA-VDR transfection into preadipocytes at 4 days. (**C**) BODIPY and (**D**) Oil Red O staining of preadipocytes after siRNA-VDR transfection of porcine intramuscular preadipocytes at 4 days. ** *p* < 0.01.

To explore the role of VDR in adipogenesis and its regulation of *SELENBP1*, we transfected porcine intramuscular preadipocytes with siRNA targeting *VDR* (si-VDR). VDR knockdown led to a significant reduction in the expression of *VDR*, *SELENBP1*, and various adipocyte markers, including *PPARγ*, *ADIPOQ*, and *PLIN1*, on day 4 of adipogenic differentiation (*p* < 0.01; Figure 8A,B). Additionally, BODIPY and Oil Red O staining demonstrated that VDR knockdown significantly decreased lipid droplet accumulation in the cells (Figure 8C,D). These results suggest that VDR plays a pivotal role in regulating the differentiation of porcine intramuscular preadipocytes into adipocytes by modulating *SELENBP1* expression, ultimately influencing lipid accumulation during adipogenesis.

## 3. Discussion

Fat deposition plays a crucial role in both livestock production and human health, being linked to conditions such as obesity, insulin resistance, and cardiovascular diseases [21,22]. Thus, understanding the molecular mechanisms underlying porcine IMF development holds value not only for enhancing pork quality but also for offering insights into human-related metabolic diseases. Adipogenesis, particularly adipogenic differentiation, is a multi-step process governed by a complex network of transcription factors and signaling pathways [23]. Previous studies have identified key regulators of adipogenesis, such as *PPARγ* and *C/EBPα*, which play critical roles in differentiation [24,25,26]. Despite these findings, adipogenic differentiation has been less explored from the perspective of chromatin accessibility in pigs. In the present study, we employed ATAC-seq and RNA-seq methodologies to provide an integrated analysis of chromatin accessibility and gene expression during adipogenic differentiation. Our findings reveal that VDR regulates IMF deposition in pigs by modulating *SELENBP1* transcription. This newly established regulatory network offers novel insights into the transcriptional control of IMF development.

We generated a comprehensive map of epigenomic changes during the adipogenic differentiation of porcine intramuscular preadipocytes by examining chromatin accessibility at day 0 and day 4. Our analysis identified significant changes in chromatin accessibility, with 63,479 peaks being upregulated and 2681 peaks being downregulated between the Pread and Ad groups. These changes correspond to 20,198 upregulated and 822 downregulated genes, respectively. Of note, the Ad group displayed an elevated ATAC signal within a ±3 kb region of the TSS compared with the Pread group, suggesting increased chromatin accessibility near these regulatory regions during adipogenic differentiation. In a study on yak intramuscular adipogenesis, chromatin accessibility significantly increased within a ±2 kb range of TSS sites during adipogenic differentiation [27]. Comparable results have been observed during muscle development and embryonic growth, supporting the hypothesis that increased chromatin accessibility around TSS regions is associated with genome activation during differentiation [28,29]. Furthermore, on comparing porcine intramuscular preadipocytes at different adipogenic differentiation stages, we observed that lipid droplets start emerging on day 4 of induction, corresponding with a significant increase in chromatin accessibility, especially around the TSS regions. This suggests a potential link between chromatin remodeling and genome activation during adipocyte development. Motif enrichment analysis of the chromatin peaks revealed the enrichment of key transcription factors, including C/EBPβ, CTCF, AP-1, FRA1, and ATF3, among the upregulated peaks. These transcription factors play crucial roles in adipogenesis. For instance, C/EBPβ is known to activate the transcription of C/EBPα and PPARγ, two critical regulators that enhance the expression of downstream adipogenic genes, thus driving adipocyte formation [30,31]. Additionally, CCCTC-binding factor (CTCF) has been shown to co-localize with activating transcription factor 4 (ATF4) at the promoters of key adipogenic genes, further activating their expression [32]. Activator protein-1 (AP-1) knockout studies have demonstrated its role in promoting adipocyte differentiation and apoptosis, thus reducing body weight and fat accumulation [33]. Fibroblast growth factor 2 (FGF-2) also contributes to adipogenesis, as FGF-2 activates the miR-29a/SPARC signaling pathway via the transcriptional upregulation of FOS-related antigen 1 (FRA1), promoting intramuscular adipogenesis in human skeletal muscle [34]. Conversely, activating transcription factor 3 (ATF3) acts as a negative regulator of adipogenesis, suppressing fat formation and enhancing lipolysis through the AMPK and extracellular regulated kinase (ERK) pathways [35,36].

The integration of ATAC-seq and RNA-seq data revealed significant correlations between chromatin accessibility and gene expression, identifying 1750 genes with a notable overlap. Among these, 1374 genes showed elevated chromatin accessibility peaks in their promoter regions, suggesting a transcriptional regulatory mechanism driving adipogenesis. This is consistent with prior results that emphasized transcriptional regulation as a primary driver of adipogenesis [37,38]. GO term and KEGG pathway analyses of these co-upregulated genes indicated enrichment in lipid metabolism, fatty acid metabolism, PPAR signaling pathway, and insulin resistance. Phospholipase A/acyltransferase 3 (*PLAAT3*) inactivation has been linked to insulin resistance and reduced adipocyte differentiation via the PPARγ signaling pathway in human adipose stem cells [39]. AMPK is recognized as a key regulator of energy balance and can suppress adipogenesis by controlling lipid metabolism [40]. In the proteomic analysis of LDM of Xidu black pigs, 56 proteins exhibited increased expression in the high-IMF group compared with the low-IMF group [20]. We further analyzed chromatin accessibility of the promoter regions and mRNA expression levels of 56 upregulated proteins. Of these, 12 genes, including *PLIN1*, fatty acid-binding protein 4 (*FABP4*), *FASN*, apolipoprotein E (*APOE*), and *ADIPOQ*, demonstrated significant increases in both chromatin accessibility and transcriptional levels. These genes are known to play critical roles in adipogenesis and meat quality [41,42,43,44,45]. Notably, ATAC-seq analysis specifically highlighted the upregulation of chromatin accessibility in the promoter region of *SELENBP1* following adipogenic induction, further strengthening the role of transcriptional regulation in IMF deposition. These findings substantially boost our understanding of the transcriptional regulatory networks governing IMF deposition.

In this study, *SELENBP1* was markedly upregulated during adipogenic differentiation, and its knockdown significantly hindered the process. Studies have demonstrated that *SELENBP1* is upregulated in mature adipocytes and serves as a marker for adipocyte differentiation and maturation [46]. The gene is also involved in lipid metabolism, with its knockout affecting the *PPARα* signaling pathway in mouse kidneys [47]. In addition to its transcriptional role, SELENBP1 has been identified as having enzymatic activity that converts methanethiol into hydrogen peroxide (H_2_O_2_), hydrogen sulfide (H_2_S), and formaldehyde [48]. Both H_2_O_2_ and H_2_S are crucial signaling molecules in adipogenesis, aiding the transition from preadipocytes to mature adipocytes [49,50]. Interestingly, the addition of H_2_S donors has been shown to partially mitigate the reduction in lipid accumulation caused by *SELENBP1* knockdown in 3T3-L1 cells [51]. These findings underscore the essential role of *SELENBP1* in adipogenesis and highlight its potential as a key regulatory factor in IMF deposition and fat metabolism.

ATAC-seq analysis revealed three upregulated peaks in the *SELENBP1* promoter region following adipogenic induction, thereby prompting further study of the transcriptional regulatory mechanisms governing this gene. The results demonstrated that the VDR can bind to the promoter region of *SELENBP1*, subsequently promoting its expression and affecting adipogenic differentiation of preadipocytes. VDR-deficient mice exhibited a systemic lack of *VDR*, leading to a decrease in adipose tissue mass, an elevation in overall energy expenditure, and an improved resistance to high-fat-diet-induced obesity [52]. Notably, VDR knockout mice aged 8 months exhibited significantly smaller adipocyte sizes compared to their wild-type counterparts, alongside symptoms such as alopecia and heightened energy expenditure [53]. Furthermore, VDR-deficient stem cells demonstrated impaired adipogenesis, and a *VDR* antagonist inhibited lipogenesis in mesenchymal progenitor cells [54]. By contrast, visceral adipose tissue mass was elevated in mice with adipose-tissue-specific *VDR* knockout [55]. These findings indicate that *VDR* is integral to adipogenesis regulation and exerts differential effects on various adipocyte differentiation stages.

## 4. Materials and Methods

### 4.1. Cell Isolation, Culture, and Adipogenic Differentiation

The intramuscular preadipocyte cells were isolated using previous methodologies [56]. Intramuscular preadipocyte cells were isolated from the LDM of 3-day-old Duroc–Landrace–Yorkshire piglets under sterile conditions. The LDM tissue was first thoroughly washed with a 0.9% sodium chloride solution, followed by treatment with phosphate-buffered saline (PBS) (Gibco, Waltham, MA, USA). The adipose tissue was finely diced into approximately 1 mm^3^ fragments for further processing. The cells were isolated through enzymatic digestion by using type II collagenase (2 mg/mL) (Sigma, St. Louis, MO, USA). The digestion was conducted at 37 °C for 120 min with continuous stirring in a water bath. The resulting cell suspension was filtered through a series of nylon mesh filters (100, 70, and 40 μm) to remove larger tissue debris. The resulting filtrate was then centrifuged at 1000 rpm for 5 min to pellet the cells. These pellets were resuspended in DMEM-F12 medium (Gibco) supplemented with 10% fetal bovine serum (Gibco) and cultured at 37 °C in a 5% CO_2_ atmosphere. For adipogenic differentiation, confluent cells were treated with an induction medium containing 0.5 mmol/L of 3-isobutyl-1-methylxanthine (Sigma), 1 μmol/L of dexamethasone (Sigma), and 5 μg/mL of insulin (Sigma). After 2 days, the induction medium was replaced with a maintenance medium containing 5 μg/mL insulin (Sigma), which was refreshed every 2 days until day 8 [57].

### 4.2. Cell Transfection, Plasmids, and Luciferase Assay

Before transfection, the cells were cultured until they reached 70%–80% confluence. siRNA was transfected into the cells using Lipofectamine RNAiMAX (Invitrogen, Carlsbad, CA, USA), and plasmids were introduced using Lipofectamine 3000 (Invitrogen). Seven truncated fragments of the *SELENBP1* promoter region in pigs were inserted into the *pGL3-basic* vector (Promega, Madison, WI, USA). The primers used to amplify these fragments are listed in Table S9. For the luciferase assays, porcine intramuscular preadipocytes were plated in 24-well plates. To normalize the transfection efficiency, the cells were transfected with 10 ng/well of *pRL-TK* (Promega). After 4 days of adipogenic differentiation, luciferase activity was measured using a PerkinElmer 2030 Multilabel Reader (PerkinElmer, Waltham, MA, USA).

### 4.3. RNA Interference

siRNAs targeting *SELENBP1*, *VDR*, and a negative control (siRNA-NC) were designed and synthesized (GenePharma, Suzhou, China). Their sequences are as follows: siRNA-SELENBP1 sense sequence: 5′-GCACCAAGUCACGCACCAAdTdT-3′; siRNA-VDR sense sequence: 5′-CCAACACGCUGCAGACCUAdTdT-3′.

### 4.4. Oil Red O and BODIPY Staining

In the Oil Red O staining protocol, cultured cells were initially rinsed two times with PBS to remove any residual media and subsequently fixed with 4% paraformaldehyde (Sigma) for 10 min at room temperature. Following fixation, the cells were stained with a working solution of Oil Red O for 30 min. For BODIPY staining, the cells were subjected to a similar fixation process with 4% paraformaldehyde, and intracellular lipids were visualized after staining with 0.5 nM BODIPY™ 493/503 (Invitrogen) for 10 min. The cells were examined and photographed under a Nikon microscope (Nikon, Tokyo, Japan).

### 4.5. Quantitative Real-Time PCR

Total cellular RNA was extracted using TRIzol (Invitrogen) reagent. Table S10 lists the sequences of primers used in quantitative real-time PCR (qRT-PCR). Complementary DNA (cDNA) was synthesized using the cDNA Synthesis Kit (Thermo Fisher Scientific, Waltham, MA, USA), and Oligo(dT)18 primers were used to initiate the synthesis. qRT-PCR was performed using the QuantStudio 6 Flex Real-Time PCR System (Applied Biosystems, Foster City, CA, USA) and the iTaq Universal SYBR Green Supermix (Bio-Rad, Richmond, CA, USA). All qRT-PCR reactions were performed in triplicate, and gene expression levels were normalized to β-actin expression by using the 2^−ΔΔCt^ method.

### 4.6. Western Blotting

RIPA lysis buffer (Beyotime, Shanghai, China) was used to extract cellular protein. Subsequently, the proteins were separated through SDS-PAGE, and a Mini Trans-Blot Cell (Bio-Rad) was employed to transfer the proteins onto polyvinylidene fluoride membranes (Millipore, Billerica, MA, USA). Immunoblotting was performed with primary antibodies targeting SELENBP1 (1:2000; ABclonal, Wuhan, China, A1222), PPARγ (1:1000; Cell Signaling Technology, Beverly, CA, USA, 2435S), PLIN1 (1:500; ABclonal, A16295), ADIPOQ (1:1000; ABclonal, A2543), GAPDH (1:5000; ABclonal, AC002), and VDR (1:100; Cell Signaling Technology, 12550). Protein expression was detected using an Image Quant LAS4000 mini (GE Healthcare Life Sciences, Piscataway, NJ, USA).

### 4.7. ChIP

ChIP was performed using the EZ-ChIP™ Kit (Millipore). Cells were sonicated using the AVCX130 system (Sonics & Materials, Newtown, CT, USA). For immunoprecipitation, anti-VDR (Cell Signaling Technology, 12550) and anti-IgG (ABclonal, AS126) were used. DNA obtained from the immunoprecipitated complex was amplified through qRT-PCR. Table S10 lists the primer sequences employed.

### 4.8. RNA-Seq

Libraries satisfying the established quality criteria were pooled and sequenced on Illumina platforms using the PE150 strategy (Novogene, Beijing, China). The raw sequencing reads were assessed using FastQC (v0.11.9), with high-quality clean reads being generated using Trimmomatic (v0.39). These clean reads were aligned to the Sus scrofa 11.1 reference genome by using HISAT2 (v2.2.1). DEGs were identified using the “DESeq2” R package (v1.36.0) and by applying the criteria of |log2(fold change)| ≥ 1 and a false discovery rate-adjusted *p* value of <0.05. Three biological replicates were used in this analysis.

### 4.9. ATAC-Seq

ATAC-seq was performed according to established protocols [15]. ATAC-seq libraries were generated and purified using AMPure beads, and their quality was assessed using a Qubit (Thermo Fisher Scientific) to ensure the accuracy of DNA quantification. Initial quality control of the raw sequencing reads was performed with FastQC. Then, Fastp (v0.19.11) software was used to eliminate adapters and low-quality sequences. Using Burrows–Wheeler Alignment software (version 0.7.12), The sequencing data were aligned to the Sus scrofa 11.1 genome. SAM files were converted to the BAM format by using SAMTools (v1.6), which were then employed for peak calling. Peak calling was performed using MACS2 (version 2.2.6) to identify open chromatin regions, thereby applying the parameters (-q 0.05 –call-summits –nomodel –shift -100 –extsize 200). A peak was defined based on a q-value threshold of < 0.05. The “ChIPseeker” R package (v1.16.1) was used to analyze peak distribution across various genomic regions. BigWig files were visualized using IGV (v2.18.2). The DiffBind (v3.6.5) tool was employed to evaluate peak differences among groups, adhering to the following criteria: |log2(fold change)| ≥ 1 and *p* < 0.05. Additionally, the HOMER findMotifsGenome.pl tool was employed for identifying transcription-factor-binding motifs within chromatin peak regions. The analysis included three biological replicates.

### 4.10. Gene Annotation and Functional Enrichment Analyses

The functional analysis of DEGs and DARs was performed by using the results of the GO terms and KEGG enrichment pathway analyses. These analyses were executed using the “clusterProfiler” R package (version 4.4.4). A significance threshold of *p* < 0.05 was established for identifying relevant GO terms and pathways.

### 4.11. Statistical Analysis

All results are expressed as mean values along with their respective standard deviations. A two-tailed *t*-test was conducted to compare the two groups, with each treatment condition replicated three times. Significant differences were assigned using an independent-samples *t*-test. *p* < 0.05 was denoted as a significant difference (*), and *p* < 0.01 was classified as a highly significant difference (**).

## 5. Conclusions

To the best of our understanding, this study is the first comprehensive examination of the regulatory network linking chromatin accessibility to gene expression during the adipogenic differentiation of porcine intramuscular preadipocytes. Adipogenic differentiation was found to be associated with the transcriptional regulation of numerous adipogenesis-related genes. Additionally, we unveiled a novel regulatory mechanism governing this process through the VDR–SELENBP1 axis.

## Figures and Tables

**Table 1:** Summary of ATAC-seq data.

Sample	Raw Reads	Raw Bases	Clean Reads	Clean Bases	Clean Ratio	Q20	Q30
Pread_1	43,273,445	12.98	42,091,064	10.17	78.35%	96.23%	90.67%
Pread_2	41,588,977	12.48	40,947,178	9.85	78.93%	95.66%	88.92%
Pread_3	46,146,628	13.84	44,687,427	11	79.48%	96.04%	90.27%
Ad_1	45,324,817	13.6	44,699,441	10.4	76.47%	97.40%	93.00%
Ad_2	43,628,308	13.09	43,057,977	9.75	74.48%	97.62%	93.52%
Ad_3	49,397,933	14.82	48,753,889	10.98	74.09%	97.60%	93.49%

**Table 2:** Summary of RNA-seq data.

Sample	Raw Reads	Raw Bases	Clean Reads	Clean Bases	Q20	Q30
Pread_1	82,917,056	12.44 G	77,760,288	11.66 G	96.72%	92.10%
Pread_2	83,103,868	12.47 G	78,304,090	11.75 G	96.73%	92.14%
Pread_3	91,072,606	13.66 G	85,485,102	12.82 G	96.69%	92.00%
Ad_1	81,312,684	12.2 G	75,767,820	11.37 G	96.21%	91.03%
Ad_2	84,770,750	12.72 G	80,038,420	12.01 G	96.57%	91.77%
Ad_3	80,076,524	12.01 G	72,053,888	10.81 G	96.21%	91.00%

## Data Availability

The data that support the findings of this study are available from the corresponding authors upon reasonable request.

## Supplementary Materials

The following supporting information can be downloaded at: https://www.mdpi.com/article/10.3390/ijms252312528/s1.
