# Supplementary material for: Integration of ATAC-Seq and RNA-Seq Reveals VDR–SELENBP1 Axis Promotes Adipogenesis of Porcine Intramuscular Preadipocytes
Source: Int J Mol Sci. 2024 Nov 22;25(23):12528. doi: 10.3390/ijms252312528 (PMC11641700; doi:10.3390/ijms252312528)
Supplement: Supplementary file 1 [file ijms-25-12528-s001.zip › Figure S3.pdf]

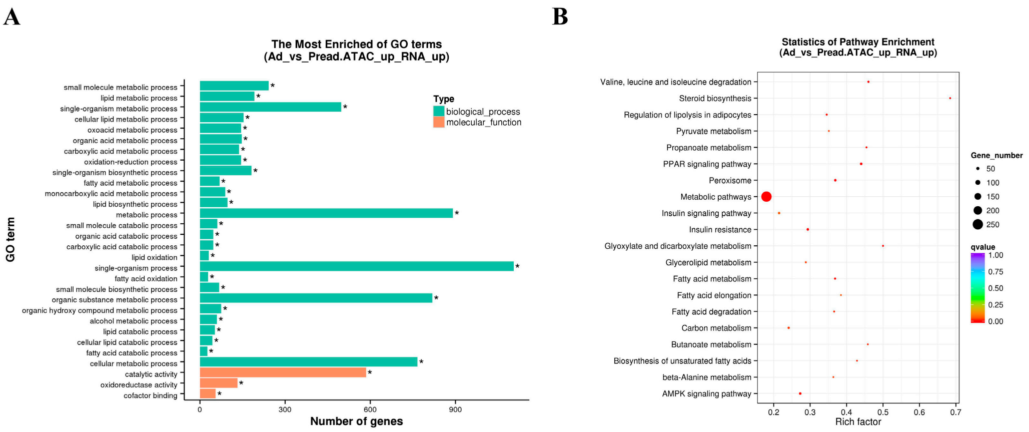

Figure S3. GO and KEGG analysis of differential peaks. (A) GO enrichment analysis of overlapping differential genes. Red represents biological processes, green represents cellular components, and blue represents molecular functions. (B) KEGG enrichment analysis of overlapping differential genes.*$p < 0.05$.
