# Supplementary figures and images for: Integration of ATAC-Seq and RNA-Seq Reveals VDR–SELENBP1 Axis Promotes Adipogenesis of Porcine Intramuscular Preadipocytes
Source: Int J Mol Sci. 2024 Nov 22;25(23):12528. doi: 10.3390/ijms252312528 (PMC11641700; doi:10.3390/ijms252312528)

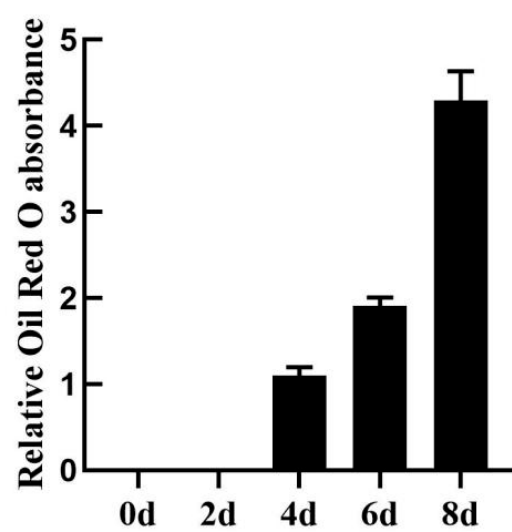

**Figure S1.** The results of quantification of the oil red O staining.

Supplement: Supplementary file 1 [file ijms-25-12528-s001.zip › Figure S1.pdf]

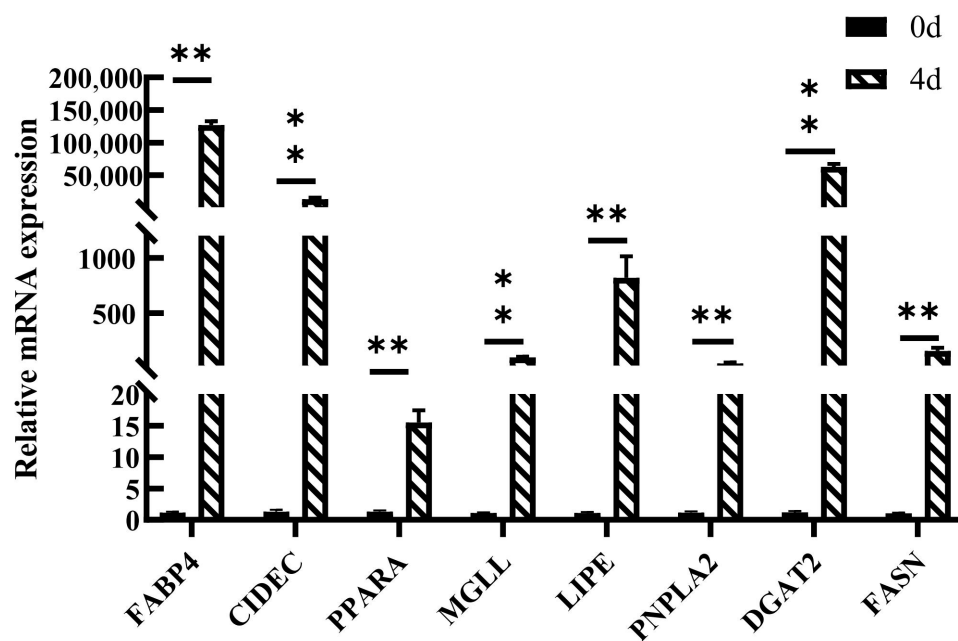

**Figure S2.** Verification of RNA-seq data by RT-qPCR. \*\* $p < 0.01$ .

Supplement: Supplementary file 1 [file ijms-25-12528-s001.zip › Figure S2.pdf]
